# Scientific Discoveries in Germany

**Published:** 1837-04

**Authors:** 


### SCIENTIFIC DISCOVERIES IN GERMANY.

(*Extract of a Letter from* Professor Weber, *of Leipzig, dated Dec.* 1836.)

At the Association of Naturalists at Jena, which I attended last September, a very interesting discovery (made by M. Fischer, of Carlsbad, and confirmed by M. Ehrenberg, of Berlin,) was communicated,—viz. that *slate* (Polierschiefer), and likewise *flint* (Feuersteine), in great part consist of the shells or envelopes of petrified animalcules. Several species of animalcules have an envelope which contains silica, and therefore resists the action even of a glowing temperature. I have myself examined several varieties of slate; and, on comparing the animalcules I therein found with corresponding ones now in a living state, I satisfied myself that the same descriptions really occur very numerously in the above mineral. Ehrenberg has reversed the experiment, by evaporating (to dryness) mud in which these animalcules abound, and then burning it in a porcelain stove. He thus produced an artificial stone, which, on being examined, was found to contain the envelopes (Panzer) of the animalcules in the identical state in which they manifested themselves in the natural mineral.

Another remarkable discovery was that of M. Göppert, of Breslau,—viz. that portions of plants, steeped in a metallic solution, imbibe the latter to repletion. If afterwards submitted to a glowing heat, the metal becomes reduced, and represents the precise form of the portion of plant, down to its minutest parts. The same experiment may be made with earthy solutions, and an artificial petrifaction thus be obtained.

You will remember being with us at the St. James's Hospital, when my brother and I proved, by experiment on the dead subject, that the head of the femur is retained within the acetabulum, or socket, *not* by the ligaments and muscles, but by the power of atmospheric pressure.* To these experiments we have since made an addition. Having sawed out the entire acetabulum, together with the upper part of the femur, (without injuring the ligaments,) from a dead body, we suspended the whole portion, by the acetabulum, from the lid of the bell appertaining to an air-pump, and attached a weight of two pounds to the femur. On exhausting the bell to within two or three inches, the head of the femur sinks to the extent of half an inch. On then readmitting the air, the head rises back again into the socket, despite the weight with which it is clogged. The same phenomenon manifests itself when circular division of the capsular ligament has been previously made; in which case, the upper half of that ligament performs the office of a valve. If the weight attached to the femur far exceed two pounds, the head of the bone delapses to the utmost extent that the ligamentum teres will admit of.

---

### ROYAL MEDICAL SOCIETY OF EDINBURGH.

* See an account of the experiment, as made in Germany, in our third Number, p. 236.—Ed.

